# Characterization of polysaccharides from different species of brown seaweed using saccharide mapping and chromatographic analysis

**DOI:** 10.1186/s13065-020-00727-w

**Published:** 2021-01-11

**Authors:** Shengqin Chen, Malairaj Sathuvan, Xiao Zhang, Wancong Zhang, Shijie Tang, Yang Liu, Kit-Leong Cheong

**Affiliations:** 1 Guangdong Provincial Key Laboratory of Marine Biotechnology, STU-UNIVPM Joint Algal Research Center, Institute of Marine Sciences, Shantou University, Shantou, 515063 Guangdong People’s Republic of China; 2 Department of Plastic Surgery and Burn Center, Second Affiliated Hospital, Shantou University Medical College, Shantou, Guangdong China

**Keywords:** Brown seaweed, Polysaccharide, Saccharide mapping, Chromatographic analysis, Antioxidant activity

## Abstract

Brown seaweed polysaccharides (BSPs) are one of the primary active components from brown seaweed that has a range of pharmaceutical and biomedical applications. However, the quality control of BSPs is a challenge due to their complicated structure and macromolecule. In this study, saccharide mapping based on high-performance liquid chromatography (HPLC), multi-angle laser light scattering, viscometer, and refractive index detector (HPSEC-MALLS-Vis-RID), and Fourier transform infrared (FT-IR) were used to discriminate the polysaccharides from nine different species of brown algae (BA1-9). The results showed that BSPs were composed of β-D-glucans and β-1,3−1,4-glucan linkages. The molecular weight, radius of gyration, and intrinsic viscosity of BSPs were ranging from 1.718 × 10^5^ Da to 6.630 × 10^5^ Da, 30.2 nm to 51.5 nm, and 360.99 mL/g to 865.52 mL/g, respectively. Moreover, α values of BSPs were in the range of 0.635 to 0.971, which indicated a rigid rod chain conformation. The antioxidant activities of BSPs exhibited substantial radical scavenging activities against DPPH (1,1-diphenyl-2-picrylhydrazyl) and ABTS (2, 2’-azino-bis-3-ethylbenzothiazoline-6-sulfonic acid) radicals, which indicated that the use of BSPs might be a potential approach for antioxidant supplements. Thus, this study gives insights about the structure-function relationship of BSPs, which will be beneficial to improve the quality of polysaccharides derived from marine algae.

## Introduction

Brown seaweed is a potentially renewable resource in the marine environment. It is one of the significantly farmed alga species along with the Asia coast, which has been used in traditional medicine for a long time [1]. As an excellent source of bioactive compounds such as carotenoids, dietary fibre, protein, essential fatty acids, vitamins, and minerals, brown seaweed is potentially exploited as functional ingredients for both human and animal health applications [2, 3]. This seaweed contains a large amount of carbohydrate as structural, storage, and functional polysaccharide, with the total carbohydrate content ranging from 50 to 60% of dry weight [4].

Brown seaweed polysaccharides (BSPs) have become the focus of interest. They are known to be an excellent resource with valuable biological activities finding applications as functional foods, pharmaceuticals, and cosmeceuticals. These polysaccharides pose an extensive array of biological activities, most notably antioxidant, anti-viral, anti-cancer, prebiotics, and immune-modulatory effects [5–7]. Structural features of polysaccharides such as molecular weight (*M*_*w*_), glycosidic linkages, and chain conformation, play an important role that affects their functional property [8, 9]. To promote further exploitation and full utilization of BSPs for functional food and pharmaceutical applications, a basic understanding of BSPs’physicochemical properties and biological activities need to be investigated.

The linkage type or feature analysis of BSPs is conventionally done by two-step characterisation process, the first involving gas chromatography mass-spectrometry analysis that requires samples to be methylated by derivatization followed by further confirmation by one dimension and two dimensions of nuclear magnetic resonance [10]. Owing to the complexities associated with these techniques, it is necessary to develop a specific and straight forward method to characterise the polysaccharides derived from different species of brown seaweed. Recently, specific enzymatic digestion called saccharide mapping has been proved to be a valuable technique for the identification of polysaccharides from natural resources [11]. Saccharide mapping based on enzymatic hydrolysis has been used for comparison of polysaccharides derived from natural and cultured *Cordyceps sinensis* [12] and different species of *Panax* [2]. As the polysaccharides present in different species of brown seaweed as well as marine algae is still unclear, saccharide mapping could be the right choice for comprehensive profiling of enzymatic hydrolysates of BSPs.

In the present study, “one-factor-at-a-time” approach was implemented to optimize PMP (1-phenyl-3-methyl-5-pyrazolone) conditions (PMP concentration, reaction time, and temperature of PMP derivates) on oligosaccharide derivatization, in order to improve the detection efficiency of HPLC based on PMP derivatization. Then saccharide mapping based on chromatographic analysis was developed to characterise and compare the polysaccharides in different species of brown seaweed. Besides, the *M*_*w*_, radius of gyration, intrinsic viscosity, and chain conformation of BSPs were investigated by high-performance size-exclusion chromatography coupled with multi-angle laser light scattering, viscometry, and refractive index detector (HPSEC-MALLS-Visc-RID). Furthermore, the antioxidant activities of BSPs were also investigated. We propose that the procedures used in this study could give more information about the structural and functional relationship of BSPs.

## Material and methods

### Materials and reagents

Nine batches of dried brown algae spices (BA1-9) were collected from different areas in China (Table 1). The corresponding author identified them, and the voucher specimens were deposited at the Institute of Marine Sciences, Shantou University, China.

**Table 1:** Summary of the investigated samples of various brown seaweed species and the yield of polysaccharides

Code	Species	Sources	Yield (%)
BA1	*Saccharina japonica*	Lianyungang City, Jiangsu Province	11.17
BA2	*Saccharina japonica*	Yantai City, Shandong Province	5.85
BA3	*Saccharina japonica*	Jining City, Shandong Province	6.71
BA4	*Saccharina japonica*	Ningde City, Fujian Province	8.19
BA5	*Undaria pinnatifida*	Yantai City, Shandong Province	8.36
BA6	*Undaria pinnatifida*	Lianyungang City, Jiangsu Province	9.47
BA7	*Undaria pinnatifida*	Dalian City, Liaoning Province	11.43
BA8	*Sargassum fusiforme*	Wenzhou City, Zhejiang Province	9.62
BA9	*Sargassum hemiphyllum*	Jining City, Shandong Province	12.30

Isomalto-oligosaccharides, 1-phenyl-3-methyl-5-pyrazolone (PMP), α-Amylase-1,4-α-D-Glucan-glucanohydrolase from *hog pancreas*, cellulase-1,4-β-D-glucan glucanohydrolase from *Aspergillus sp.*, and pectinase-α-1,4-glycosidic from *Aspergillus aculeatus* were purchased from Sigma (St. ouis, MO, USA). Fucosidase-α-(1–2,3,4,6)-L-fucosidase from *Homo sapiens* and lichenase-1,3(4)-β-Glucanase from *Bacillus sp.* were purchased from Megazyme (Wicklow, Ireland). All the other reagents were of analytical grade.

### Preparation of polysaccharides

The low *M*_*w*_ compounds were removed by treating 10 g pulverised brown algae samples with 200 mL methanol/dichloromethane/water (4: 2: 1; *v/v/v*). The mixture was placed on a shaking incubator at room temperature for 24 h. The precipitate obtained after centrifugation (4000×*g*, 15 min) was dried in an oven at 50 °C. The dried material was resuspended in distilled water, and kept at room temperature for 10 min. Next, the samples were heated at 90 °C on a water bath for 2 h, centrifuged (4000×*g*, 15 min), resuspended in 3-fold volumes of ethanol (95%) and then stored at 4 °C overnight. After centrifugation (4000×*g*, 15 min), the precipitate was collected and dissolved in hot water, freeze-dried for a day to obtain crude brown seaweed polysaccharides (BSPs). The yield of BSPs (%) was calculated by following equation:

$$\text{Yield of BSPs (\%)} =\frac{\text{weight of crude BSPs (g)}}{\text{weight of dried material (g)}}\times100$$

### Optimization of PMP on oligosaccharide derivatization

To identify the optimized condition of oligosaccharides-PMP derivatives, several conditions were performed by changing one factor at a time while keeping the others constant. The experimental variables were PMP concentration, reaction time, and temperature of PMP derivates.

Before derivatization, isomalto-oligosaccharides (5 mg) were dissolved in distilled water (1 mL) to get standard sample solution. High-performance liquid chromatography (HPLC) based oligomers detection of PMP derivatives was performed according to a reported method with slight modifications [13]. Briefly, the standard sample solution (50 µL) was mixed with 0.1 mol/L NaOH solution (50 µL) and 0.025-0.3 mol/L PMP methanolic solution (100 µL). The mixture was reacted at 40–90 °C for 40–140 min, and washed three times with dichloromethane, then the aqueous layer was filtered through a 0.45 µm membrane. The analysis of PMP-labeled samples was carried on an Agilent 1200 HPLC system (Alltech, USA), equipped with a vacuum degasser, a binary pump, an autosampler, and a diode array detector (DAD). The analytical column was a VisionHT C18 column (250 mm × 4.6 mm, 5 µm) operated at 30 ℃. The injection volume was 20 µL. Elution was at a flow rate of 0.8 mL/min. The mobile phase A consisted of acetonitrile and mobile phase B was 0.1 mol/L phosphate buffer at pH 6.7, using an isocratic elution of 16% A and 84% B.

### Saccharide mapping based on chromatographic analysis

BSPs samples were dissolved in water at a concentration of 5 mg/mL. Sample solutions (250 µL) were incubated for 12 h at 40 °C with the following enzyme (500 µL), respectively: cellulase, α-amylase, lichenase, pectinase, and α-fucosidase at a final concentration of 20 U/mL. Subsequently, the mixtures were heated at 80 °C for 20 min to stop the reaction. The hydrolysates were derived with optimized PMP conditions and then analysed by HPLC as described above.

### Monosaccharide composition

Monosaccharide composition of BSPs was determined by 1-phenyl-3-methyl-5-pyrazolone (PMP) pre-column derivatization described as a published method [13]. The sample was analyzed by high performance liquid chromatography (HPLC) system (Shimadzu, Japan) equipped with a VisionHT C18 column (250 mm × 4.6 mm, 5 µm) and a UV detection at 245 nm.

### Fourier transforms infrared spectroscopy (FT-IR)

The FT-IR (MAGNA-IR 750, Thermo Nicolet Co., USA) spectrum was determined in the frequency range of 4000 − 400 cm^− 1^ by pressing BSPs samples (1 mg) and KBr (100 mg) into a pellet.

### HPSEC-MALLS-Visc-RID

The *M*_*w*_, polydispersity, radius of gyration, and intrinsic viscosity [*η*] of BSPs samples were determined on high-performance size exclusion chromatography coupled with multi-angle laser light scattering, viscometer, and refractive index detector (HPSEC-MALLS-Vis-RID) system equipped with an Agilent 1100 series LC/DAD system connected with P.L.aqua gel-OH Mixed-H column (300 mm × 7.8 mm, Agilent Technologies, USA) [14]. The procedure used to measure the [*η*] of BSPs followed the classic Huggins-Kramer equations. The enzymatically digested products (1 mL) were filtered through a 0.22 µm filter membrane, and then 50 µL of the filtrate was injected into the column, before being eluted with 0.1 mol/L NaNO_3_ at a flow rate of 0.5 mL/min. The column temperature was 35 °C. Data acquisition and analysis were carried out using the ASTRA 5.0 software.

### Antioxidant activities

#### DPPH radical scavenging activity

A previously published protocol with slight modifications [15], was referred for evaluating DPPH radical scavenging efficacy of BSPs. BSPs samples (1 mL) at different concentrations were added into 1 mL DPPH solution dissolved in dehydrated alcohol (0.004%, *w/v*), then shaken and incubated for 30 min in a dark place. The absorbance values of BSPs were measured at 517 nm. DPPH radical scavenging efficacy (%) was calculated through the equation as previously described [16].

#### ABTS radical scavenging activity

The activity of BSPs samples in scavenging ABTS radicals was evaluated as described earlier, but with some minor modifications [17]. To generate ABTS radicals, a 5 mL ABTS solution (7 mmol/L) was mixed with 88 µL K_2_S_2_O_8_ (149 mmol/L), and the reaction mixture was incubated for 16 h (dark). Then, 1 mL of this solution was added to 10 µL of BSPs samples at various concentrations, and the absorbance was measured at 734 nm. ABTS radical scavenging activity (%) was calculated as previously described [17]. The 50% inhibitory concentration (IC_50_) was defined as the concentration of BSPs samples that inhibited DPPH and ABTS radical formation by 50%. The IC_50_ values were calculated from the regression equations evaluated from the concentration of samples, and the percentage of inhibition for each system was calculated.

## Results and discussion

### Effect of PMP on the enzymatic products oligosaccharide derivation

Since carbohydrates have no UV absorbance, the reagents PMP is the most common labels that derivate with reducing sugars under mild conditions, which provides a high yield of single derivatives. A wealth of information is available on optimisation for single derivatives, but studies on the oligosaccharide derivatization were rarely reported. Accordingly, the PMP method, as reported so far, is not uniform for oligosaccharide measurements. Therefore, an optimised PMP-derivatization about oligosaccharide derivatization was developed. The condition of PMP derivates was optimised based on the ‘one-factor-at-a-time’ approach to optimise the condition of PMP concentration, reaction time, and temperature of PMP derivates.

To explore the optimised conditions of oligosaccharides-PMP derivatives, the standards of isomalto-oligosaccharides were initially investigated as models for the optimisation of PMP derivatization. The results were the sum of the peak area of oligosaccharides-PMP derivative detected by HPLC-DAD.

**Fig. 1:**
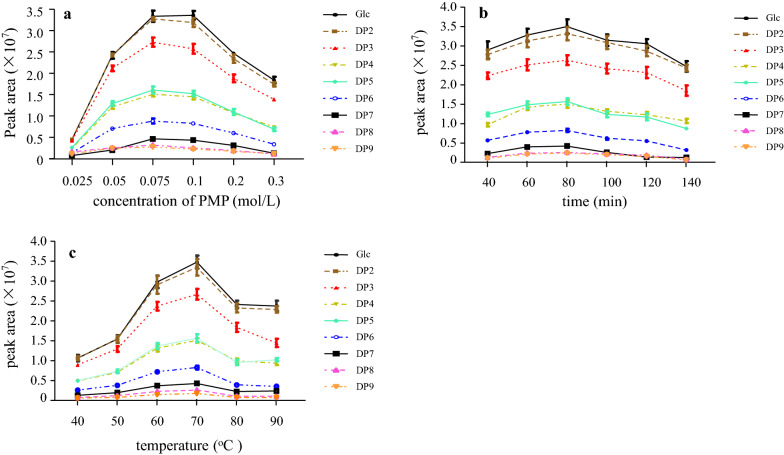
Optimization of oligosaccharides-PMP derivation. **a** The concentration of PMP. **b** Reaction temperature. **c** Reaction time

The product of oligosaccharide-PMP derivative could be influenced by the concentration of PMP, reaction time, and temperature of the derivatization. Therefore, the effect of various conditions on the derivatization potency was optimised by changing one argument at a time while keeping other arguments steady. To further research and display the optimised conditions of the above-mentioned oligosaccharide-PMP derivative, a line chart has been plotted, which distinctly reveals the effects of reciprocity of the independent variables. The results of the concentration of PMP, reaction time, and temperature of derivatization effect were shown in Fig. 1.

To investigate the PMP concentration effect on derivatization, a series of tested reactions with oligosaccharide as a model saccharide were performed. The result of the effect of reaction PMP concentration (0.025, 0.05, 0.075, 0.1, 0.2, and 0.3 mol/L) on total peak area (proportional to the yield of the derivative) were presented in Fig. 1a. The yield of the total peak area increased with the increase of the PMP concentration within a range from 0.025 to 0.075 mol/L. Performing the reaction at PMP concentration from 0.075 to 0.1 mol/L showed little effect. For a concentration ranging from 0.1 to 0.3 mol/L, the total peak area was negatively correlated with PMP concentration. Therefore, performing the labelling routine at 0.075 mol/L was recommended. The peak area of oligosaccharide at 0.075 mol/L and 0.1 mol/L PMP concentrations was similar; however, we recommend a working limit of 0.075 mol/L, which is in agreement with previous publications. When detecting carbohydrate-PMP derivatives, Rühmann et al. [18] considered that 0.075 mol/L PMP concentrations revealed to be optimal condition to minimise system peaks and enhance effectiveness.

To define the optimal amount of reaction time on the PMP derivatization reaction, a series of derivatization experiments with reaction time were carried out. As shown in Fig. 1b, the highest total peak area of oligosaccharide was obtained at 80 min. A low or high reaction time decreased the total peak area of oligosaccharide, as low reaction time reduces the efficiency of oligosaccharide-PMP derivatization, and high reaction time lead to oligosaccharide degradation. The effect of reaction time on oligosaccharide-PMP derivatization was in accordance with Han et al. [19] HPLC analysis of chitosan-PMP derivatives revealed the reaction time at 60 min was the optimum derivatization condition of oligosaccharide and which was close to our results.

Besides the concentration of PMP and reaction time, other parameters affecting the enzymatic products of oligosaccharide derivation using PMP were examined, and the results showed that temperature profoundly affected the PMP derivative which was determined by total peak area of oligosaccharides (Fig. 1c). The total peak area almost linearly upgrades below 70 °C; however, a decline was noted above this point due to partial decomposition of the reaction product at this condition. Based on the maximum peak area of derivative, the optimal reaction temperature was found to be 70 °C. These results are in accordance with Wang et al.., who concluded that the optimal condition based on response surface methodology for the glucose-PMP derivatization was between 70 and 73 °C for 120–140 min [20]. Therefore, it is recommended to perform the labelling at 70 °C.

### Characterisation of BSPs on the basic of saccharide mapping

Brown seaweed polysaccharides (BSPs) were extracted by using hot water based on our previous work [21]. As shown in Table 1, the yield of BSPs ranged from 5.85 to 12.30%. The highest yield of polysaccharide (12.30%) was BA9, which obtained from *Sargassum hemiphyllum*.

**Fig. 2:**
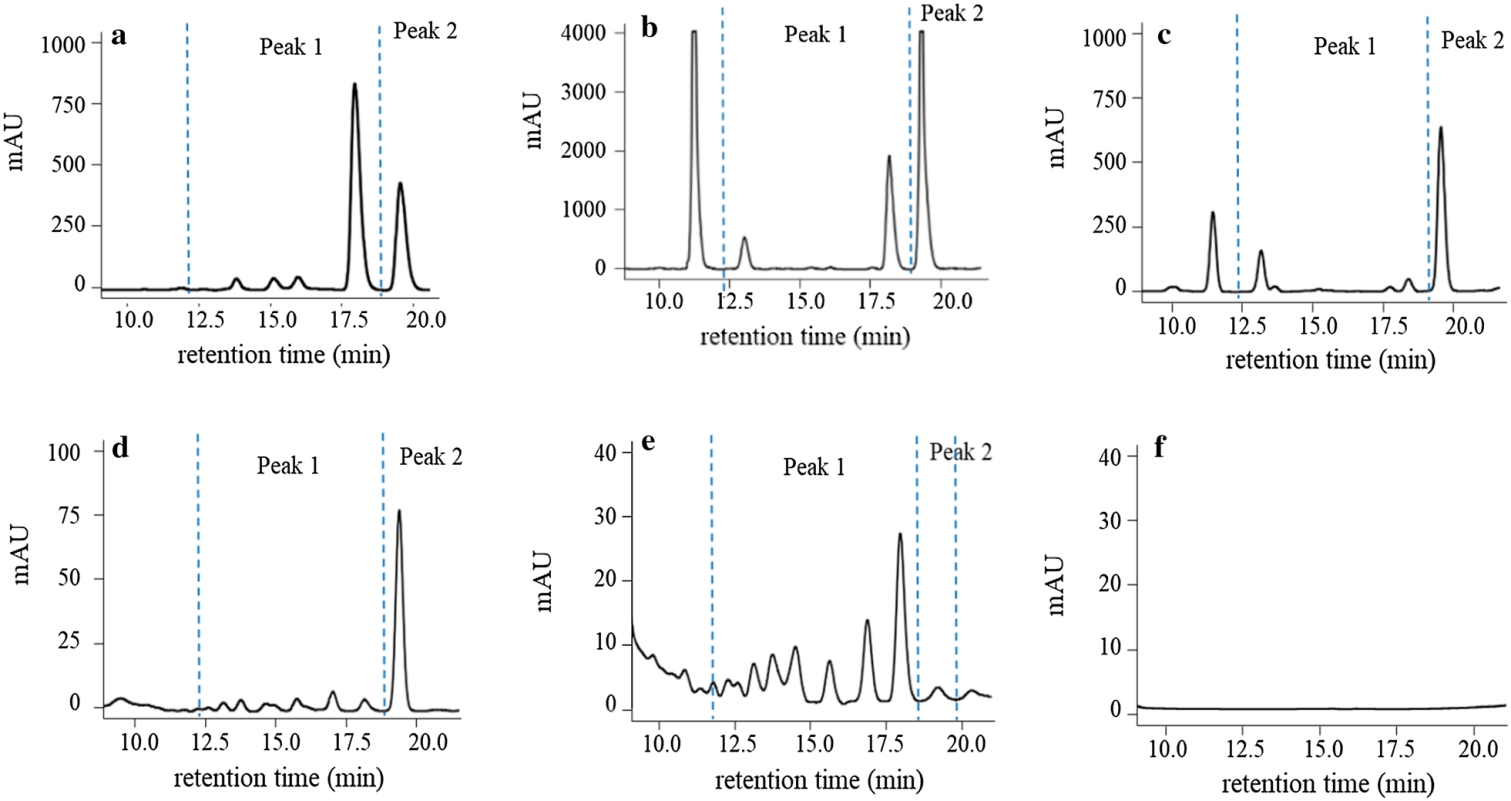
Typical HPLC chromatogram of saccharide mapping of brown seaweed polysaccharides, sample BA1. **a** Amylase; **b** pectinase; **c** cellulase; **d** lichenase; **e** fucosidase; **f** blank. Peak 1 area: oligomers, Peak 2: monosaccharide

After enzymolysis, HPLC chromatogram of BSPs showed two main peaks. As shown in Fig. 2, peak 1 represented oligomers, and peak 2 indicated monosaccharide. Lichenase are glycosyl hydrolases that comprise of structurally diverse groups of enzymes that act on the β-glycosidic bonds between polysaccharides. Cellulase catalyzes the hydrolysis of β-1,4-glucosyl linkages, while lichenase catalyzes the hydrolysis of β-1,4-glucosyl linkages adjacent to a β-1,3-glucosyl linkage on the non-reducing end of β-1,3−1,4 type of polysaccharides [22]. The typical HPLC chromatogram of saccharide mapping of BSPs by cellulase and lichenase were showed in Fig. 2c and d, respectively. All the BSPs samples (BA1-BA9) showed positive responses to cellulase and lichenase (Table 2), which indicated all polysaccharides samples were composed of β-D-glucans and β-1,3−1,4 type of polysaccharides. The β-1,3−1,4-glucan, mostly found in the plant and fungi, has a peculiar distribution pattern among eukaryotes. More recent studies point out that some BSPs can build up β-1,3−1,4-glucan chains in their cell wall, for example, *Monodus subterraneus*, *Laminaria hyperborean*, and *Saccharina latissima* [23].

α-Amylase is a hydrolase enzyme that catalyzes the hydrolysis of internal α-1,4-glycosidic linkages, while pectinase randomly attacks the α-1,4-glycosidic linkages of the polysaccharides chains [24]. All investigated seaweed samples (BA1–BA9) showed a positive response to α-amylase and pectinase (Table 2), and the typical chromatograms are shown in (Fig. 2a, b), which indicated that these samples contained the α-D-glycosidic linkages. The results are similar to Li et al. [25], who reported that bioactive polysaccharides extracted from *Sargassum pallidum* had a 1-4-glycosidic linkage.

**Table 2:** Saccharide mapping response of brown seaweed polysaccharides derived from brown algae

Samples	Enzyme				
	Amylase	Pectinase	Cellulase	Lichenase	Fucosidase
BA1^a^	A +^b^	A+	A+	A+	A+
BA2	A+	A+	A+	A+	A+
BA3	A+	A+	A+	A+	A+
BA4	A+	A+	A+	A+	A+
BA5	A+	A+	A+	A+	A+
BA6	A+	A+	A+	A+	A+
BA7	A+	A+	A+	A+	A+
BA8	A+	A+	A+	A+	A+
BA9	A+	A+	A+	A+	A+

^a^The sample codes were the same as Table 1

^b^Positive response

The α-fucosidase can catalyze the hydrolysis of fucose from fucoidan, *via* cleavage of α-1,3-L-fucopyranose, α-1,4-L-fucopyranose, and possibly also of α-1,2-L-fucopyranose in fucoidan [26]. All investigated samples showed a positive response to α-fucosidase (Table 2). Typical chromatograms were shown in (Fig. 2e), which indicated that those samples contain fucoidan type of polysaccharides. According to Wu et al. [27], fucoidan is structurally made of the repetitive units of α-1,3-L-fucopyranose and α-1,4-linked L-fucopyranose.

### Monosaccharide composition of different BSPs

The mole percentages of monosaccharides were summarized in Table 3. The results showed that polysaccharides in BSPs mainly consist of mannose, glucuronic acid, glucose, galactose, and fucose, with small amounts of rhamnose and arabinose. In BA1-4, included 8.3–12.2% of mannose, 11.7–14.5% of glucuronic acid, 12.1–23.4% of galactose, and 48.7–62.4% of fucose. This is consistent with previous study by Zhang et al. [21], results showed that polysaccharides extracted from *Saccharina japonica* were mainly composed of mannose, glucuronic acid, galactose, and fucose in a molar ratio of 1.0: 1.2: 3.6: 4.1. Other polysaccharides in BA5-7 contained glucose up to 55.6–74.0%, indicating that polysaccharides from *Undaria pinnatifida* were mainly composed of glucose. In BA8 and BA9, the monosaccharides consist of mannose, glucuronic acid, glucose, galactose, and fucose, with fucose up to 35.1–43.7%. The results suggested that brown seaweed polysaccharides from different species exhibited different monosaccharide compositions.

**Table 3:** Monosaccharide composition of polysaccharides from brown seaweed polysaccharides samples

Samples	Man (%)	Rha	GlcA (%)	GalA	Glc (%)	Gal (%)	Ara	Fuc (%)
BA1	10.3	0.7%	13.8	–	1.4	23.4	1.7%	48.7
BA2	11.2	1.4%	13.4	–	1.9	19.4	1.9%	50.7
BA3	12.2	0.8%	14.5	–	0.6	21.2	1.8%	48.9
BA4	8.3	3.8%	11.7	–	1.6	12.1	0.1%	62.4
BA5	7.0	–	13.7	–	55.6	11.8	–	11.9
BA6	4.4	–	8.6	–	74.0	6.3	–	6.8
BA7	5.2	–	10.1	–	68.1	8.3	–	8.3
BA8	10.8	–	11.4	–	17.4	25.3	–	35.1
BA9	10.3	–	12.2	–	6.8	27.0	–	43.7

*Man *Mannose, *Rha *Rhamnose, *GlcA *Glucuronic acid, *GalA *Galacturonic acid, *Glc *Glucose, *Gal *Galactose, *Ara *Arabinose, *Fuc *Fucose, – not detected

### FT-IR of different BSPs

**Fig. 3:**
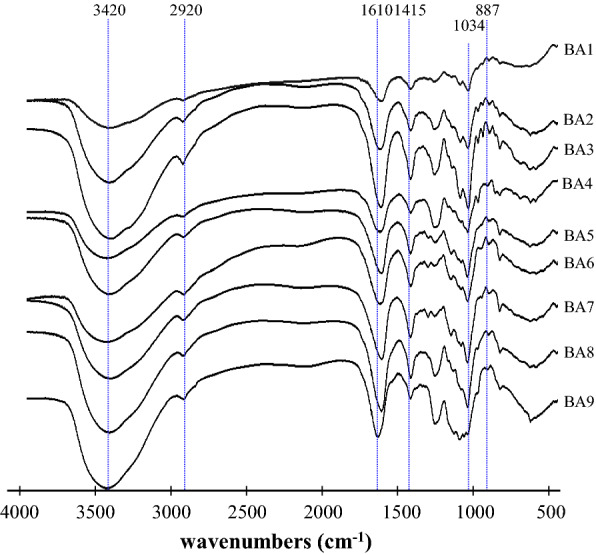
FT-IR spectra of brown seaweed polysaccharides samples. The codes of polysaccharide samples were the same as in Table 1

FT-IR spectroscopy is one of the important analytical techniques extensively used to study the molecular structures and the conformations of macromolecules to identify the vibrations between the different atoms in molecules. The spectrum obtained between 400 and 4000 cm^− 1^ can be used to analyse the structural features of polysaccharides, including glucosidic bonds and functional groups. Functional characteristics and structural features of BSPs were analysed using FT-IR. As shown in Fig. 3, the sharp band at 887.76 cm^− 1^ (C–S–O) suggested a pattern of sulfate substitution [28]. The band at 1034 cm^− 1^ was ascribed to uronic acid by the C-O stretching vibration [29]. The absorption peak presented at 1415 cm^− 1^ was assigned to the C=O stretching mode of the fucoidan [30]. The strong absorption band at 1240–1255 cm^− 1^ (S=O) stretching confirmed the existence of a significant amount of sulfate in the polysaccharides [31]. The band at 1250 cm^− 1^ and 1028 cm^− 1^ was attributed to the presence of asymmetric O=S=O stretching vibration of a sulfate group [32]. Previous report showed that signals at 1610–1635 cm^− 1^ were assigned to the asymmetric strength vibration of COO- of uronic acids [33]. The major absorption band at 3420 cm^− 1^ represents to O-H group [34].

### Conformation features of different BSPs

HPSEC-MALLS-Visc-RID has demonstrated to be a powerful technique for determining the *M*_*w*_, [*η*], chain conformation features of polysaccharides without using a series of dextran standards [35]. In otherwords, the HPSEC chromatograms are helpful to determine physical properties of polysaccharides, which are in turn beneficial to discriminate and improve the quality of polysaccharides derived from natural resources. In this study, the *M*_*w*_, [*η*], the radius of gyration (*R*_*g*_), and chain conformation of BSPs samples were investigated by HPSEC-MALLS-Visc-RID.

**Fig. 4:**
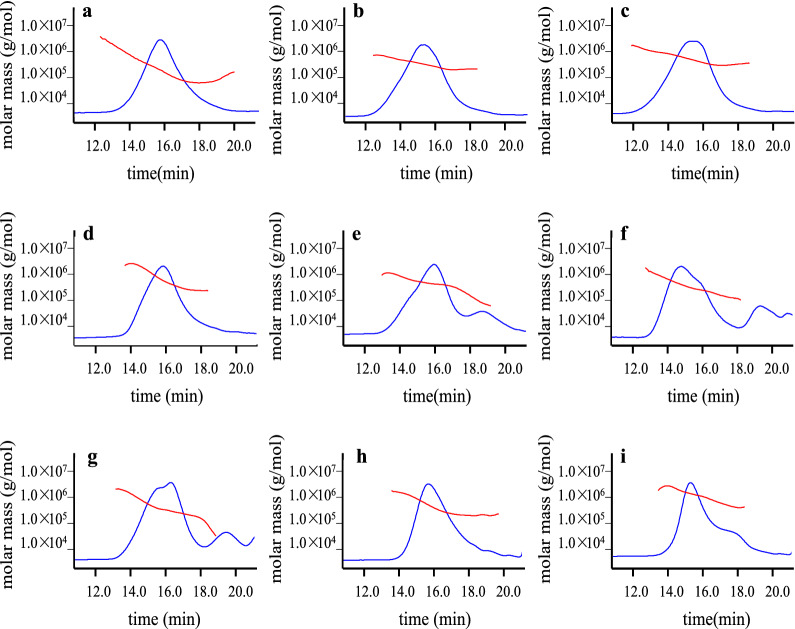
HPSEC chromatograms (solid lines) with molecular weight distribution (dots) of the brown seaweed polysaccharides samples **a** BA1, **b** BA2, **c** BA3, **d** BA4, **e** BA5, **f** BA6, **g** BA7, **h** BA8, **i** BA9 determined by multi-angle laser light scattering, viscometer, and refractive index detector. The codes of samples were the same as in Table 1

The *M*_*w*_ distributions of BSPs analysed on the RID were shown in Fig. 4, while the values of [*η*] were obtained from viscometer. The values of *M*_*w*_ and *R*_*g*_ of BSPs were investigated by MALLS according to the Zimm method based on Rayleigh-Gans-Debye theory for light scattering using the following equation:

$${\raise0.7ex\hbox{${Kc}$} \!\mathord{\left/
 {\vphantom {{Kc} {{R_\theta }}}}\right.\kern-\nulldelimiterspace}
\!\lower0.7ex\hbox{${{R_\theta }}$}} = {\raise0.7ex\hbox{$1$} \!\mathord{\left/
 {\vphantom {1 {{M_w}}}}\right.\kern-\nulldelimiterspace}
\!\lower0.7ex\hbox{${{M_w}}$}}\left[ {1 + \left( {{\raise0.7ex\hbox{${16{\pi ^2}{n^2}}$} \!\mathord{\left/
 {\vphantom {{16{\pi ^2}{n^2}} {3{\lambda ^2}}}}\right.\kern-\nulldelimiterspace}
\!\lower0.7ex\hbox{${3{\lambda ^2}}$}}} \right)R_g^2{{\sin }^2}\left( {{\raise0.7ex\hbox{$\theta $} \!\mathord{\left/
 {\vphantom {\theta  2}}\right.\kern-\nulldelimiterspace}
\!\lower0.7ex\hbox{$2$}}} \right)} \right] + 2{A_2}c$$

where the optical constant is represented as *K *= [4*π*^2^*n*^2^(*dn*/*dc*)^2^]/(*N*_*A*_*λ*^4^); *C* is the polysaccharides concentration; *R*_*θ*_ is the Rayleigh ratio; *λ* is the wavelength; *n* is the refractive index of the solvent (0.9% NaCl aqueous solution); *N*._*A.*_is Avogadro’s number, and *A*_2_ is the second virial coefficient. The MALLS instrument was calibrated using filtered (0.2 µm, Millipore) HPLC-grade toluene, and normalised using a dextran standard (25 kDa, low polydispersity). All the obtained results are summarised in Table 4.

**Table 4:** Molecular weight, intrinsic viscosity, and chain conformation of the investigated brown seaweed polysaccharides samples

Samples	*M*_*w*_(Da)	*R*_*g*_(nm)	[*η*] (mL/g)	α
BA1	1.718 × 10^5^ (± 0.407%)	32.5 (± 0.1%)	386.75 (± 0.75%)	0.689
BA2	3.663 × 10^5^ (± 0.655%)	44.5 (± 0.1%)	704.26 (± 0.47%)	0.971
BA3	4.276 × 10^5^ (± 0.943%)	51.5 (± 0.1%)	729.62 (± 0.46%)	0.635
BA4	6.630 × 10^5^ (± 0.690%)	49.6 (± 0.1%)	389.39 (± 0.69%)	0.700
BA5	2.803 × 10^5^ (± 0.506%)	43.8 (± 0.1%)	865.52 (± 0.44%)	0.827
BA6	2.949 × 10^5^ (± 0.408%)	48.8 (± 0.0%)	708.46 (± 1.10%)	0.884
BA7	2.438 × 10^5^ (± 0.469%)	39.3 (± 0.1%)	816.83 (± 0.41%)	0.888
BA8	1.909 × 10^5^ (± 0.239%)	30.2 (± 0.1%)	406.01 (± 1.25%)	0.663
BA9	5.136 × 10^5^ (± 0.426%)	32.2 (± 0.1%)	360.99 (± 1.85%)	0.652

*M*_*w*_: Molecular weight; *R*_*g*_: radius of gyration; [*η*]: intrinsic viscosity; α: exponent of Mark-Houwink-Sakurada equation. The sample codes were the same as Table 1.

The *M*_*w*_ of BSPs samples were determined ranging from 1.718 × 10^5^ Da to 6.630 × 10^5^ Da. It was observed that there was a molecular weight difference in the investigated seaweed samples, which may be due to the disparity of brown algae in species and their growth condition [36]. Similarly, Ali et al. [37] also reported that the molecular weight of brown algae *Stypocaulon scoparium* polysaccharide was determined as 2.236 × 10^5^ Da. The BA1, BA4, BA8, and BA9 samples have relatively low [*η*] values ranging from 360.99 to 406.01 mL/g, while the BA2, BA3, BA5, BA6, and BA7 samples have high [*η*] values, which were determined to be in the range of 704.26–865.52 mL/g. The relative low [*η*] values were in agreement with water-soluble polysaccharides isolated from the Tunisian brown seaweed *Cystoseira compressa* with the [*η*] of 400 mL/g [38]. Similarly, the relative high [*η*] values were also in accordance with polysaccharide extracted from *Cystoseira trinode*, *Sargassum latifolium*, and *Cystoseira myrica* in the range of 860–870 mL/g [39].

Moreover, the values of *R*_*g*_ and *M*_*w*_ were then utilised to establish the double logarithmic relationships of *M*_*w*_–*R*_*g*_ based on the following equations:

$${\text{R}}_{\text{g}}=\text{k}{\left({\text{M}}_{\text{w}}\right)}^{{\upalpha }}$$

According to the theory of macromolecules solution, the exponent α values of 0.33, 0.50 to 0.60, and 1.0 reflected chain shape corresponding to spheres, random coils, and rigid rods, respectively [40]. It also gives access to information on the conformation of the polysaccharides. The exponent α values of the polysaccharides from brown algae was calculated to be in the range of 0.635 to 0.971. These results indicated that the BSPs adopted a rigid rod. Khajouei et al. [41] reported that the exponent α values of polysaccharides derived from *Nizimuddinia zanardini* as 0.64 that corresponds to the rod conformation.

### BSPs possess anti-oxidant activities *in vitro*

The DPPH has a stable free radical, which has been widely accepted as a tool for estimating the free radical-scavenging activities of antioxidants. This assay is used to test the anti-oxidative ability of polysaccharide functioning as hydrogen donors. The amounts of DPPH scavenging activity of various BSPs were recorded in Table 5. Among the nine different seaweeds, BA6 (*Undaria pinnatifida*) showed a relatively higher IC_50_ with 1.28 mg/mL. Followed by BA7 (*Undaria pinnatifida*), which was 1.21 mg/mL. While the lowest IC_50_ were recorded in BA9 (*Sargassum hemiphyllum*, 0.85 mg/mL) and BA4 (*Saccharina japonica*, 0.87 mg/mL). Thus, the seaweed BA9 (*Sargassum hemiphyllum*) collected from Jining City was determined to be the richest source of antioxidant as compared to the other seaweeds tested in this study. The antioxidant capacity of fucoidan is due to the ability to donate the H atoms to form stable DPPH-H molecule [42]. The obtained results were similar to the earlier study that witnessed a reduction in the activity towards the increasing concentration of fucoidan extracted from *Spatoglossum asperum*, with an IC_50_ value of 93.69 µg/mL [43]. Similarly, *Sargassum pallidum* sulfated polysaccharide [44] and *Cystoseria barbata* polysaccharide [45] also exhibited favourable DPPH radical-scavenging activity.

ABTS assay is to determine the capability of hydrogen-donating antioxidants to scavenge ABTS radical in the solution and prevent lipid oxidation via chain-breaking antioxidants. The IC_50_ of ABTS scavenging activity of various BSPs was tabulated in Table 5. The lowest IC_50_ value (0.55 mg/mL) was observed in BA4 (*Saccharina japonica*) collected from Ningde City, followed by BA1 (*Saccharina japonica)* from Lianyungang City (IC_50_ = 0.57 mg/mL). The highest IC_50_ (0.83 mg/mL) was observed in BA7 (*Undaria pinnatifida*) obtained from Dalian City and followed by BA6 (*Undaria pinnatifida*) collected from Lianyungang City. Thus, the best hydrogen-donating ability to scavenge ABTS was observed in BA4 (*Saccharina japonica*) collected from Ningde City. Previous studies in other species of algae suggested that sulfated polysaccharide had antioxidant activities. For example, sulfated polysaccharides from *Gracialria corticata* had a maximum scavenging activity of 74.5% with a concentration of 125 µg/mL [46]. The ABTS• cation radical activity plays a vital role by H abstraction reaction, due to the chemical structure of sulfated polysaccharide [47]. The observations of the study revealed that BSPs had excellent scavenging ability on ABTS radical. The potent antioxidant activities of BSPs may represent an exciting advancement in search of novel functional applications relevant to following industrial field, including pharmaceuticals, nutraceuticals, cosmeceuticals, and functional foods.

**Table 5:** Effect of brown seaweed polysaccharides samples in scavenging DPPH and ABTS radicals.Values are given as IC_50,_ which are means of triplicate determinations

Samples	IC_50_ values (mg/mL)	
	DPPH radicals	ABTS radicals
Control (V_c_)	0.31 (± 0.91%)	0.34 (± 0.51%)
BA1^a^	0.91 (± 0.42%)	0.57 (± 1.12%)
BA2	1.12 (± 0.87%)	0.69 (± 0.13%)
BA3	0.90 (± 0.73%)	0.59 (± 0.84%)
BA4	0.87 (± 0.53%)	0.55 (± 0.46%)
BA5	1.16 (± 0.35%)	0.66 (± 0.65%)
BA6	1.28 (± 0.93%)	0.78 (± 0.52%)
BA7	1.21 (± 1.13%)	0.83 (± 0.76%)
BA8	1.05 (± 1.07%)	0.63 (± 0.43%)
BA9	0.85 (± 0.59%)	0.58 (± 1.04%)

^a^The sample codes were the same as Table 1

## Conclusion

In this study, BSPs from different species that were collected from several regions of China were characterized using a chromatographic method based on saccharide mapping. Results showed that different species of brown seaweed had similar chemical characteristics, which showed a positive response to cellulase, α-amylase, lichenase, pectinase, and α-fucosidase. All investigated BSPs samples were found to have a rigid rod confirmation features through theory of macromolecules solution. In addtion, the *M*_*w*_ and [*η*] of BSPs are vital factor in the expression of bioactivities and thus were confirmed in this study. The *M*_*w*_ of BSPs samples were in the range of 1.718 × 10^5^– 6.630 × 10^5^ Da, while [*η*] was ranging from 360.99 to 865.52 mL/g. The BSPs from different species of brown seaweed exhibited profitable antioxidant activities in terms of DPPH and ABTS radicals scavenging abilities, which revealed that BSPs could be employed as antioxidants in the food, pharmaceutical, and cosmetics industry. Therefore, this study is beneficial to improve the quality of brown seaweed as well as their performance in pharmaceutical and biomedical applications.

## Data Availability

The datasets used and/or analysed during the current study are available from the corresponding author on reasonable request.

## Abbreviations

BSPs: Brown seaweed polysaccharides
HPLC: High-performance liquid chromatography
HPSEC-MALLS-Vis-RID: Multi-angle laser light scattering, viscometer, and refractive index detector
FT-IR: Fourier transform infrared
BA1-9: Brown algae
*M*_*w*_: Molecular weight
PMP: 1-phenyl-3-methyl-5-pyrazolone
DAD: Diode array detector
η: Intrinsic viscosity
DPPH: 1,1-diphenyl-2-picrylhydrazyl
ABTS: 2, 2’-azino-bis-3-ethylbenzothiazoline-6-sulfonic acid
IC_50_: 50% inhibitory concentration
Man: Mannose
Rha: Rhamnose
GlcA: Glucuronic acid
GalA: Galacturonic acid
Glc: Glucose
Gal: Galactose
Ara: Arabinose
Fuc: Fucose
*R*_*g*_: Radius of gyration
C: Polysaccharides concentration
*R*_*θ*_: Rayleigh ratio
n: Refractive index of the solvent
*N*._*A*_: Avogadro’s number
